# The VIPR2-selective antagonist KS-133 changes macrophage polarization and exerts potent anti-tumor effects as a single agent and in combination with an anti-PD-1 antibody

**DOI:** 10.1371/journal.pone.0286651

**Published:** 2023-07-05

**Authors:** Kotaro Sakamoto, Wararat Kittikulsuth, Eijiro Miyako, Akumwami Steeve, Rika Ishimura, Shinsaku Nakagawa, Yukio Ago, Akira Nishiyama

**Affiliations:** 1 Research & Development Depertment, Ichimaru Pharcos Company Limited, Motosu, Gifu, Japan; 2 Depertment of Pharmacology, Faculty of Medcine, Kagawa University, Miki-cho, Kita-gun, Kagawa, Japan; 3 Graduate School of Advanced Science and Technology, Japan Advanced Institute of Science and Technology, Nomi, Ishikawa, Japan; 4 Center for Supporting Drug Discovery and Life Science Research, Graduate School of Pharmaceutical Science, Osaka University, Suita, Osaka, Japan; 5 Laboratory of Biopharmaceutics, Osaka University, Suita, Osaka, Japan; 6 Global Center for Medical Engineering and Informatics, Osaka University, Suita, Osaka, Japan; 7 Department of Cellular and Molecular Pharmacology, Graduate School of Biomedical and Health Sciences, Hiroshima University, Hiroshima, Hiroshima, Japan; Universite de Nantes, FRANCE

## Abstract

We have previously demonstrated that KS-133 is a specific and potent antagonist of vasoactive intestinal peptide receptor 2 (VIPR2). We have also shown that vasoactive intestinal peptide–VIPR2 signaling affects the polarity and activation of tumor-associated macrophages, which is another strategy for cancer immunotherapy apart from the activation of effector T cells. In this study, we aimed to examine whether the selective blockade of VIPR2 by KS-133 changes the polarization of macrophages and induces anti-tumor effects. In the presence of KS-133, genetic markers indicative of tumor-aggressive M1-type macrophages were upregulated, and conversely, those of tumor-supportive M2-type macrophages were downregulated. Daily subcutaneous administration of KS-133 tended to suppress the growth of CT26 tumors (murine colorectal cancer-derived cells) implanted subcutaneously in Balb/c mice. To improve the pharmacological efficacy and reduce the number of doses, we examined a nanoformulation of KS-133 using the US Food and Drug Administration-approved pharmaceutical additive surfactant Cremophor® EL. KS-133 nanoparticles (NPs) were approximately 15 nm in size and stable at 4°C after preparation. Meanwhile, KS-133 was gradually released from the NPs as the temperature was increased. Subcutaneous administration of KS-133 NPs once every 3 days had stronger anti-tumor effects than daily subcutaneous administration of KS-133. Furthermore, KS-133 NPs significantly enhanced the pharmacological efficacy of an immune checkpoint-inhibiting anti-PD-1 antibody. A pharmacokinetic study suggested that the enhancement of anti-tumor activity was associated with improvement of the pharmacokinetic profile of KS-133 upon nanoformulation. Our data have revealed that specific blockade of VIPR2 by KS-133 has therapeutic potential for cancer both alone and in combination with immune checkpoint inhibitors.

## Introduction

Vasoactive intestinal peptide receptor 2 (VIPR2) is a class-B G protein-coupler receptor that participates in various physiological function by interacting with its ligand vasoactive intestinal peptide (VIP). VIP–VIPR2 signaling has attracted attention as a drug target in the fields of central nervous system diseases, oncology, and immunity. In 2021, we generated 13-mer bicyclic peptide KS-133 as a potent and selective antagonist to VIPR2 [1]. KS-133 is resistant to protease degradation, suppresses the phosphorylation of CREB (a downstream signal of VIPR2) in brain tissues and exerts pharmacological effects in a mouse model of psychiatric disorders [1]. Several lines of evidence support associations of VIPR2 with cancer and immunity. For example, VIP–VIPR2 signaling regulates tumor cell migration [2], and inhibition of VIPR signaling promotes CD8+ T cell proliferation and immune function [3–5]. In addition, VIP is released from tumor and immune cells, and it regulates immune reactions such as anti-inflammatory activities through interactions with VIPR1 and VIPR2 [6].

In addition to the aforementioned study, we reported that VIPR2-mediated signaling contributes to the polarity of tumor-associated macrophages (TAMs) [7]. Activation of TAMs has attracted attention as another means of cancer immunotherapy involving the activation of effector T cells (e.g., CD8+ T cells) by immune checkpoint inhibitors [8–10]. It has been reported that the pharmacological effects of immune checkpoint inhibitors are not sufficient against intractable solid tumors such as colorectal and pancreatic cancers [11–13]. One reason for this finding is the tumor microenvironment, which hinders T cell infiltration into tumor tissues [14]. Meanwhile, TAMs invade deeper into tumor tissues. Although TAMs have multiple activation states, they can be roughly divided to inflammatory M1-type and anti-inflammatory M2-type phenotypes. M1-type TAMs are antigen-presenting cells that highly express class II major histocompatibility complex and activate T cell function for anti-cancer activity. However, as tumors progress, M2-type TAMs are activated by cytokines released by tumor tissues, and they secrete anti-inflammatory cytokines and support tumor growth. It was demonstrated in a clinical study that the survival rate of patients with cancer is negatively correlated with M2-type TAM infiltration into tumor tissues [8–10]. It is also known that a higher M1/M2 ratio improves the prognosis of colorectal cancer [8–10]. Thus, re-polarization of M2-type TAMs to M1-type TAMs will be beneficial for cancer treatment. As previously reported, blockade of VIPR2 signaling suppresses tumor growth by enhancing the polarization and phagocytic function of M1-type macrophages in CT26 tumor-bearing mice [7]. Thus, we expected that the selective blockade of VIPR2 by KS-133, both alone and in combination with immune checkpoint inhibitors, such as anti-PD-1 antibodies would promote cancer-immune activation and induce anti-tumor effects.

A hurdle in the development of peptides as therapeutic drugs is their low stability *in vivo*. Generally, peptides are rapidly degraded by proteases, and even if they are resistant to degradation, they are prematurely excreted from the body via renal filtration. Thus, frequent dosing is needed, reducing patients’ quality of life. KS-133 is stable in plasma for at least 24 h [1]. Unfortunately, daily dosing is required for the anti-tumor effects of KS-133 *in vivo*. A versatile method for improving the pharmacokinetics of peptides is conjugation with polyethylene glycol [15, 16], but this requires fine-tuning to ensure that the target-binding activity is not attenuated [17, 18]. Therefore, we investigated a non-conjugation strategy, termed nanoformulation technology, which has been primarily used to develop drug delivery systems (DDSs) of therapeutic molecules against intracellular targets [19, 20]. In this study, nanoformulation technology was used against the extracellular target VIPR2. Here, we examined the M2-type macrophage-inducing effects of KS-133 *in vitro* and the potent anti-tumor effects of KS-133 nanoparticles (NPs) when administered alone or combined with an anti-PD-1 antibody.

## Materials and methods

### *In vitro* gene expression evaluation of M1-like and M2-like macrophages

The detailed method and DNA primer sequences were described previously [7]. RAW264.7 murine macrophage-like cells and CT26.WT murine colorectal cancer cells (CRL-2638) were purchased from RIKEN BRC (Tsukuba, Japan) and American Type Cell Collection (Manassas, VA, USA), respectively. CT26 cells (4 × 10^6^) were grown in T75 flask with 20 mL of RPMI medium containing 10% FBS for 3 days. The culture medium (CT26-CM) was collected and centrifuged at 400 *g* for 5 min. RAW264.7 cells (2.4 × 10^5^) were incubated in 24-well plate in the presence or absence of KS-133 (KS-133 concentration = 1, 3, or 10 μM, DMSO concentration = 0.1%) with 20% CT26-CM/DMEM for 3 days. During the incubation, medium containing KS-133 was replaced every day. mRNA extraction was performed using ISOGEN (Nippon Gene, Tokyo, Japan) on day 3 after CT26-CM incubation, and cDNA was prepared. The gene expression of M1 markers (*TNF-α*, *iNOS*, *CXCL10*), M2 markers (*Mrc-1*, *IL-1rn*, *CCL22*), and *β-actin* was analyzed by real-time polymerase chain reaction (PCR) using a 7300 Fast Real-Time PCR System (Applied Biosystems, Thermo Fisher Scientific, MA, USA,) and Light Cycler Fast Start DNA Master kit (Applied Biosystems). The relative mRNA expression was determined using the 2^−ΔΔCt^ method. ΔΔCt was calculated using control group data.

### Preparation of KS-133 NPs

KS-133 and KS-133(P3S) (a substitution of proline at position three of KS-133 with serine) were synthesized at SCRUM Inc. (Tokyo, Japan) using Fmoc-based solid-phase peptide synthesis as previously reported [1]. After reverse phase–high-performance liquid chromatography (RP–HPLC) purification, structural assignment was performed by matrix-assisted laser desorption ionization–time-of-flight mass spectrometry (MS). All analytical data of peptides in this report are presented in S1 Table. Cremophor EL® (CrEL) (09727–14) was purchased from Nacalai Tesque, Inc. (Kyoto, Japan). The nanoformulation of peptide using CrEL was done by basically according to the method as previously reported [21]. First, KS-133 or KS-133(P3S) (20 mg) was dissolved in DMSO (100 μL), giving a concentration of 200 mg/mL. Next, CrEL (100 μL) was mixed with purified water (350 μL). The peptide solution was added dropwise to the stirring CrEL solution and mixed. Then, the peptide/CrEL solution made into a suspension by repeated vortex agitation and sonication at approximately 50°C. Purified water (450 μL) was added to the peptide/CrEL solution, and the same process was repeated. After repeated chilling on ice, sonication under cold water, and agitation by vortex, the solution was prepared as transparent KS-133 (20 mg/mL)/DMSO (10%)/CrEL (10%) solution and stored at 4°C until use. DMSO without peptide was used to prepare control NPs.

### Structural characterization of KS-133 NPs

Negative staining was used to observe the morphology and structure of NPs using a high-resolution transmission electron microscope (H-7600; Hitachi, Tokyo, Japan) at an acceleration voltage of 100 kV. The particle size of NPs was measured in a disposable cuvette by dynamic light scattering using Zetasizer Nano ZSP (Malvern Instruments Ltd, Malvern, UK).

### *In vivo* anti-tumor evaluation

All experimental procedures that involved animals and their care were conducted in compliance with the *Guide for the Care and Use of Laboratory Animals* [22] and ARRIVE guidelines [23, 24]. These animal experiments were approved by the Animal Care and Use Committee of Kagawa University (approval No. 21650) and conducted at Kagawa University in accordance with the guidelines. To establish subcutaneous allograft tumors, CT26 cells (1 × 10^5^) were subcutaneously injected into the left groin region of BALB/cCrSlc female mice (6 weeks old; Japan SLC, Inc., Shizuoka, Japan) under isoflurane anesthesia. KS-133 (30 mg/mL)/DMSO was diluted in D-PBS (048–29805, FUJIFILN Wako Pure Chemical Corporation, Osaka, Japan) (without calcium and magnesium) (KS-133 concentration = 0.3 mg/mL, DMSO concentration = 1%), and KS-133 (20 mg/mL)/DMSO (10%)/CrEL (10%) solution was diluted in D-PBS (KS-133 concentration = 0.3 mg/mL, DMSO concentration = 0.15%, CrEL concentration = 0.15%) and injected at 100 μL (30 μg)/mouse subcutaneously every day or every 3 days to tumor-bearing mice for 3 weeks. For anti-PD-1 monotherapy or combination treatment, GoInVivo Purified anti-mouse PD-1 (9.98 mg/mL) (CD279, Cat. #114114, BioLegend, San Diego, CA, USA) was diluted in D-PBS (antibody concentration = 1.0 mg/mL) and injected at 100 μL (200 μg)/mouse intraperitoneally three times in a week. The tumor growth rate was recorded on days 1, 7, 14, and 21 by measuring the major and minor axes with a digital caliper. Measurements were transformed into tumor volumes using the following formula: tumor volume (mm^3^)  =  major axis × minor axis^2^ × 0.5. Thereafter, animals were killed by overdose of pentobarbital sodium and tumor tissues were collected. According to the regulations established by the Animal Care and Use Committee of Kagawa University, it is recommended that the experiment be stopped from an animal welfare perspective when the tumor size exceeds 10% of the body weight, in which case the animal is euthanised and deemed dead. If the tumour was large and the animal appeared to be suffering, Ibuprofen (30 mg/kg) were used.

### *In vivo* pharmacokinetic study

These animal experiments were approved by the Animal Care and Use Committee of Osaka University (approval No. Douyaku 30-3-2) and conducted at Osaka University in accordance with the aforementioned guidelines. LC–MS/MS data were obtained using an LCMS-8060NX MS system (Shimadzu Corp., Kyoto, Japan) connected to a Nexera X3 UHPLC system (Shimadzu) equipped with a Peptide BEH C18 column (1.7 μm, 2.1 × 50 mm, Waters Corp., Milford, MA, USA). Mobile phase A was 0.1% formic acid in water, and mobile phase B was acetonitrile. The elution profile of mobile phase B was 5% isocratic between 0 to 1 min, linear gradient from 5% to 50% between 1 to 1.5 min, and increase from 50% to 75% between 1.5 to 3 min. The flow rate was 0.2 mL/min. For pharmacokinetic studies, free or nanoformulated KS-133 was administered intravenously or subcutaneously to male ICR mice at 1 nmol/g body weight. Serial blood samples were collected from the tail vein using heparinized capillary tubes at the indicated times after administration. The plasma (5 μL) obtained from each blood sample was mixed with 10 μL of 0.1% formic acid in 50% (v/v) acetonitrile/water, and subsequently, 50 μL of acetonitrile was added. After centrifugation, aliquots (50 μL) of the supernatant were mixed with equal volumes of 0.2% formic acid in water and then injected into the LC–MS/MS system. Pharmacokinetic parameters were obtained from the plasma concentration–time data by non-compartmental analysis using Phoenix WinNonlin Software (Certara Companies, NJ, USA).

### Statistical analysis

Multiple-group comparisons were made using one-way or two-way analysis of variance (ANOVA), followed by Tukey’s multiple comparison test. All statistical analyses were performed using GraphPad Prism 6 (GraphPad Software Inc., La Jolla, CA). *p* < 0.05 was considered statistically significant.

## Results

### KS-133 induced M1-type macrophages *in vitro*

**Fig 1:**
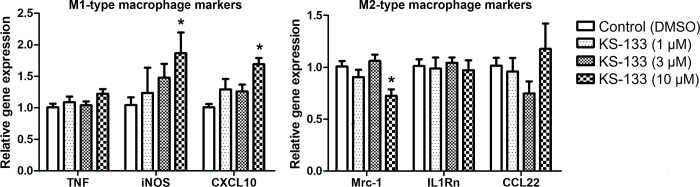
Variation of marker gene expression in M1- and M2-type macrophages in the presence of KS-133. Results are presented as the mean ± SEM (n = 3 independent experiments using separately treated cultures, *p < 0.05 vs. control by two-way ANOVA).

It has been reported that when RAW264.7 cells are incubated in the presence of the culture supernatant of CT26 cells, M1-type macrophage marker mRNA expression is decreased, and that of M2-type macrophages is increased [7]. Using this assay system, we evaluated whether KS-133 affect the mRNA expression of *TNFα*, *iNOS*, and *CXCL10* as M1-type macrophage markers and *Mrc-1*, *IL-1Rn*, and *CCL-22* as M2-type macrophage markers. As presented in Fig 1, KS-133 (10 μM) significantly enhanced the mRNA expression level of *iNOS* and *CXCL10* (*p* < 0.05). Conversely, KS-133 significantly decreased the mRNA expression of *Mrc-1* (*p* < 0.05). Therefore, VIPR2 inhibition by KS-133 changes the macrophage polarity toward M1.

### Daily subcutaneous administration of KS-133 enhanced the pharmacological effects of an anti-PD-1 antibody

**Fig 2:**
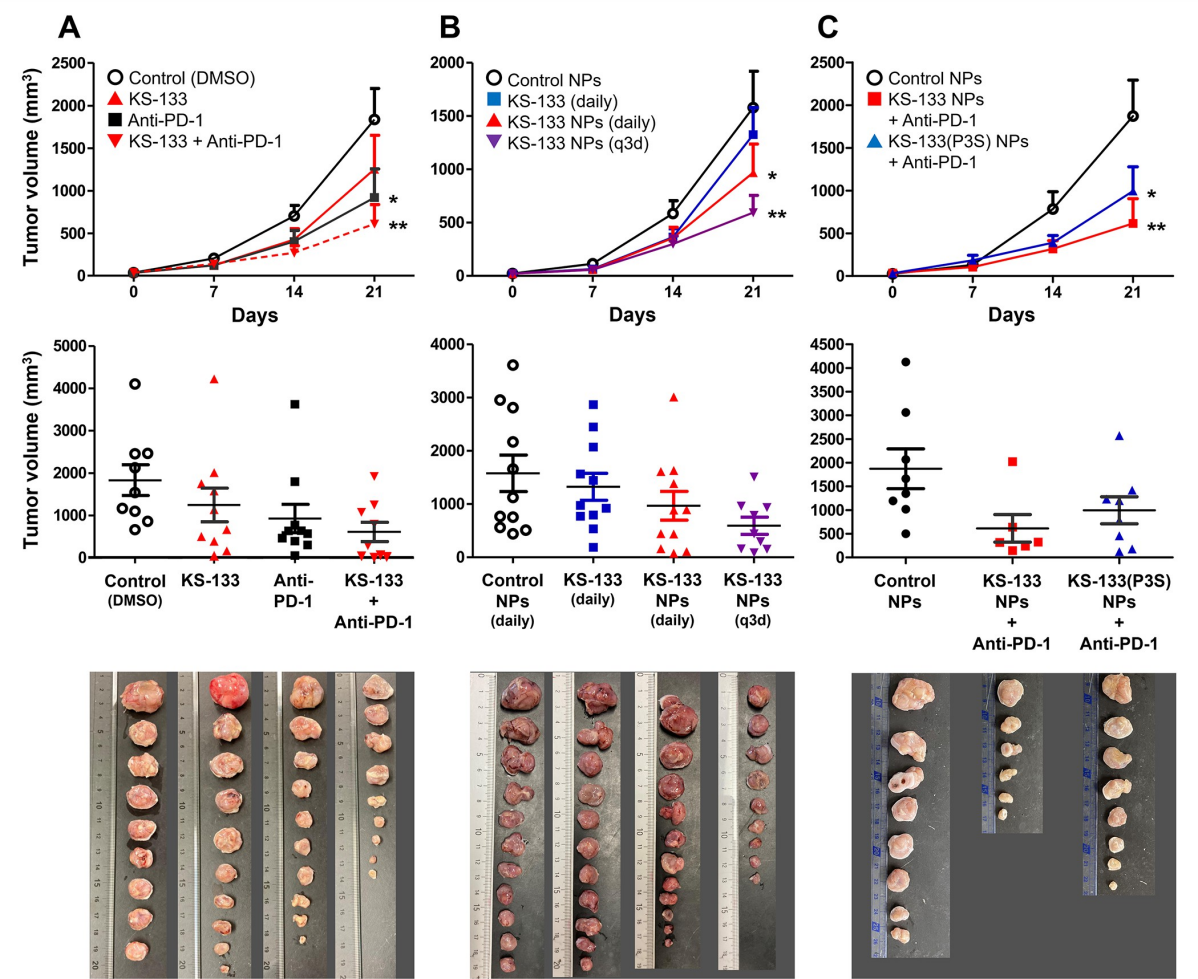
Anti-tumor effects of KS-133 and KS-133 NPs with or without anti-PD-1 antibody in mice bearing CT26 subcutaneous allografts. The mean changes in the tumor volume over time, the tumor volume at day 21, and the appearance of collected tumor samples are presented. (A) Anti-tumor efficacy of KS-133 (n = 9–10 mice per group, expressed as the mean ± SEM, *p < 0.01 and **p < 0.001 vs. vehicle control by two-way ANOVA). KS-133 (30 μg/mouse) was subcutaneously administered daily, and anti-PD-1 antibody (200 μg/mouse) was intraperitoneally administered three times in a week. (B) Anti-tumor efficacy of KS-133 NPs (n = 11 mice per group, expressed as the mean ± SEM, *p < 0.05 and **p < 0.001 vs. vehicle control by two-way ANOVA). KS-133 (30 μg/mouse) was subcutaneously administered daily, and KS-133 NPs (30 μg/mouse) were subcutaneously administered daily or once every 3 days (q3d). (C) Anti-tumor efficacy of KS-133 NPs and KS-133(P3S) NPs with an anti-PD-1 antibody (n = 6–8 mice per group, expressed as the mean ± SEM, *p < 0.01 and **p < 0.001 vs. Vehicle control by two-way ANOVA). The nanoformulated peptide (30 μg/mouse) was subcutaneously administered once every 3 days, and anti-PD-1 antibody (200 μg/mouse) was intraperitoneally administered three times in a week.

It has been reported that daily subcutaneous administration of VIPhyb, an antagonist of both VIPR1 and VIPR2, induces anti-tumor effects against CT26 cells implanted to severe combined immunodeficient (SCID) mice and enhances the pharmacological effects of an anti-PD-1 antibody [7]. We evaluated the anti-tumor effects of KS-133 alone and in combination with an anti-PD-1 antibody. As presented in Fig 2A, KS-133 alone tended to suppress CT26 tumor growth, whereas KS-133 displayed significant and additive anti-tumor effects in combination with an anti-PD-1 antibody (*p* < 0.001). Setting the tumor volume of the control group as 100%, the tumor volumes of the KS-133 monotherapy, anti-PD-1 antibody monotherapy, and KS-133/anti-PD-1 antibody combination groups were 66%, 50%, and 32%, respectively. The results in Figs 1 and 2A suggest that KS-133 can potentially be used as part of cancer immunotherapy, especially given its ability to enhance the efficacy of immune checkpoint inhibitors by inducing the polarization of M1-type macrophages. However, we found two issues. One is the poor solubility of KS-133 in aqueous solution, which makes it difficult to handle, and the other is the need for daily administration.

### Surfactants such as Tween 80 and CrEL greatly improved the water solubility of KS-133

**Fig 3:**
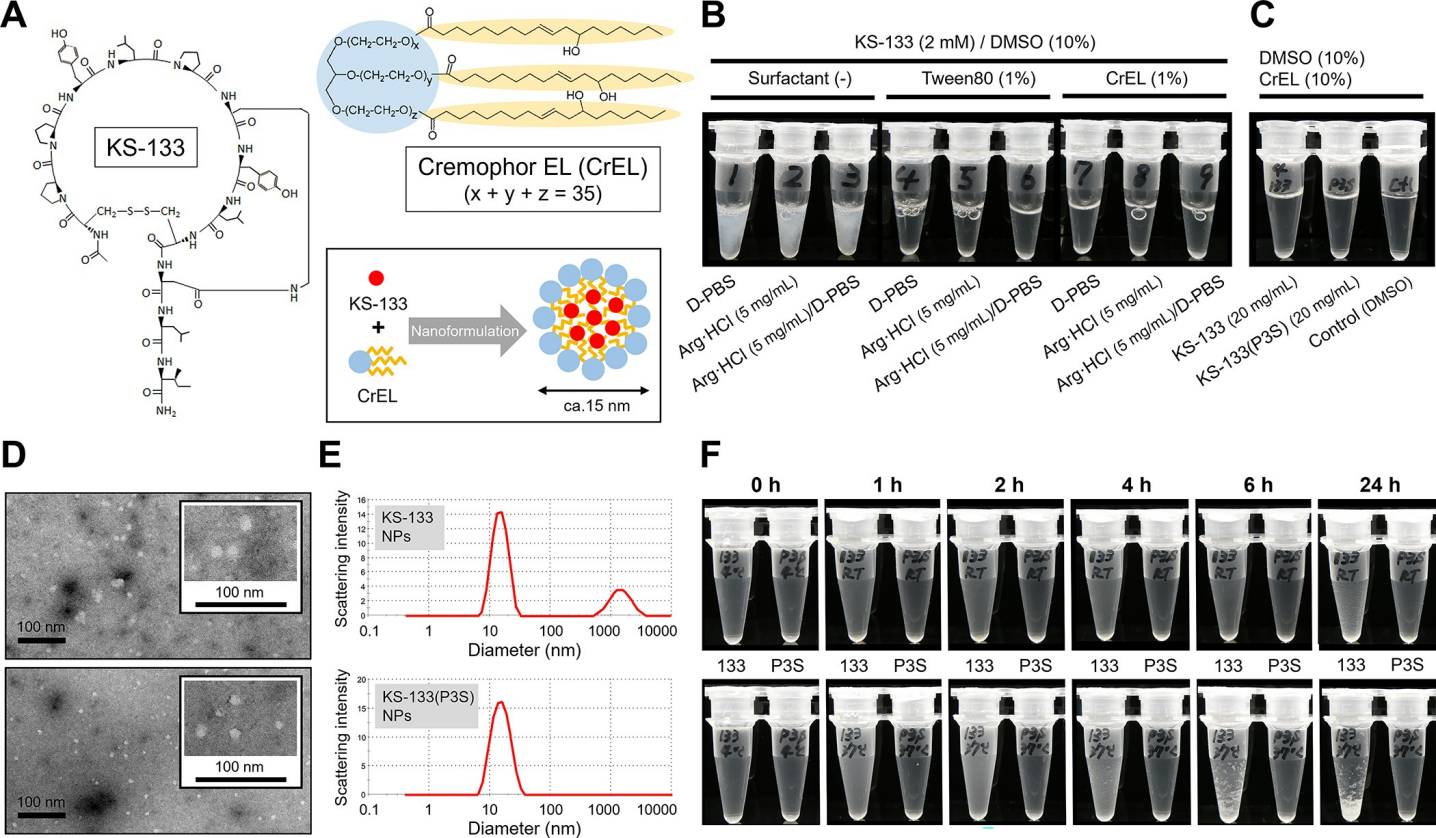
Nanoformulation of peptides using CrEL and their characterization. (A) Chemical structures of KS-133 and CrEL. In the molecular structure of CrEL, the hydrophilic moiety is highlighted in light blue, and the hydrophobic moiety is presented in orange. In the box, a schematic of the nanoformulation by mixing KS-133 and CrEL is presented. (B) Examination of the water solubility improvement of KS-133 following nanoformulation using Tween 80 or CrEL. (C) Appearance of the final nanoformulation in this study immediately after preparation. (D) Representative transmission electron microscopy analysis of KS-133 NPs (upper panel) and KS-133(P3S) NPs (lower panel). (E) Representative size distribution of KS-133 NPs (upper panel) and KS-133(P3S) NPs (lower panel). The determined particle size of KS-133 NPs was approximately 15.34 nm, and that of KS-133(P3S) NPs was approximately 15.96 nm. (F) Changes in the appearance of NP solutions at room temperature (25°C, upper panel) and at 37°C (lower panel) for 1, 2, 4, 6, and 24 h.

First, we sought to improve the water solubility of KS-133 by amino acid substitution. Using reported VIPR2/KS-133 complex model information [25], we replaced proline 3 with serine, which was expected to improve the water solubility of KS-133 without affecting its VIPR2-binding activity. Contrary to our expectations, the water solubility of KS-133(P3S) was not different from that of KS-133. Therefore, to overcome both limitations (poor water solubility and frequent dosing) simultaneously, we generated nanoformulations using the surfactants Tween 80 and CrEL, which are approved by the US Food and Drug Administration for use as pharmaceutical additives (Fig 3A). We expected that KS-133 would be incorporated into the NPs that surfactants spontaneously formed in water, improving its water solubility. As presented in Fig 3B, the addition of Tween 80 and CrEL greatly improved the aqueous solubility of KS-133. We selected CrEL due to its proven use with paclitaxel and other anti-cancer drugs [26]. Finally, KS-133 and KS-133(P3S) at a concentration of 20 mg/mL could be dissolved in DMSO(10%)/CrEL(10%) (Fig 3C).

### KS-133 was released from NPs upon increasing the temperature

The mechanism of the enhanced water solubility was considered to be the incorporation of the peptide into NPs. Transmission electron microscopy (Fig 3D) and particle size distribution analysis (Fig 3E) illustrated that NPs with a particle size of approximately 10–15 nm were formed in the solution. The prepared KS-133(P3S) NPs were transparent and stable under storage at 4°C, room temperature (25°C), and 37°C with no change in appearance (Fig 3F). The prepared KS-133 NPs were transparent and stable under storage at 4°C. However, they gradually became cloudy and precipitated as the temperature was increased. At room temperature (25°C), KS-133 NPs remained clear for several hours but displayed slight precipitation after 24 h. At 37°C, KS-133 NPs became slight cloudy after 1–2 h, exhibited slight precipitation after 4 h, and displayed visible precipitation after 6 h. The explanations could be that (1) the affinity of CrEL for KS-133(P3S) is higher than that for KS-133 (the molecular mechanism is unknown) and (2) the hydrophobic core in the NPs is tightly packed at low temperatures but moderately loosened because of molecular fluctuation at higher temperatures. Based on these explanations, KS-133, but not KS-133(P3S), was released from the NPs in a temperature-dependent manner.

### KS-133 NPs had stronger pharmacological effects with less frequent dosing

We evaluated whether KS-133 NPs exhibit anti-tumor effects *in vivo*. As presented in Fig 2B, daily subcutaneous administration of KS-133 NPs resulted in stronger pharmacological effects than daily subcutaneous administration of KS-133. Interestingly, the effects of KS-133 NPs were stronger when administered once every 3 days (q3d) than when administered daily. Setting the tumor volume of the control group at 100%, the tumor volumes of the KS-133 NPs (daily) and KS-133 NPs (q3d) groups were 60% and 38%, respectively.

The reason is unclear, but we hypothesized that CrEL was concentrated at the injection site following daily administration and the concentrated CrEL suppressed the release of KS-133 from NPs. To examine the relationship of peptide release from NPs with pharmacological efficacy and the effects of combination treatment with an anti-PD-1 antibody, an additional *in vivo* evaluation was performed (Fig 2C). The efficacy of KS-133 NPs was stronger than that of KS-133(P3S) NPs. Setting the tumor volume of the control group to 100%, the tumor volumes of the KS-133 NPs/anti-PD-1 antibody and KS-133(P3S) NPs/anti-PD-1 antibody groups were 30% and 55%, respectively. It was suggested that KS-133 NPs enhanced the pharmacological efficacy of the anti-PD-1 antibody because the tumor volume of the anti-PD-1 antibody group was 50% (Fig 2A) and that of the KS-133 NP (q3d) group was 38% (Fig 2B). Additionally, it was indicated that the extent of release of KS-133 from the NPs is related to its pharmacological efficacy.

### Nanoformulation improved the pharmacokinetic profiles of KS-133

**Fig 4:**
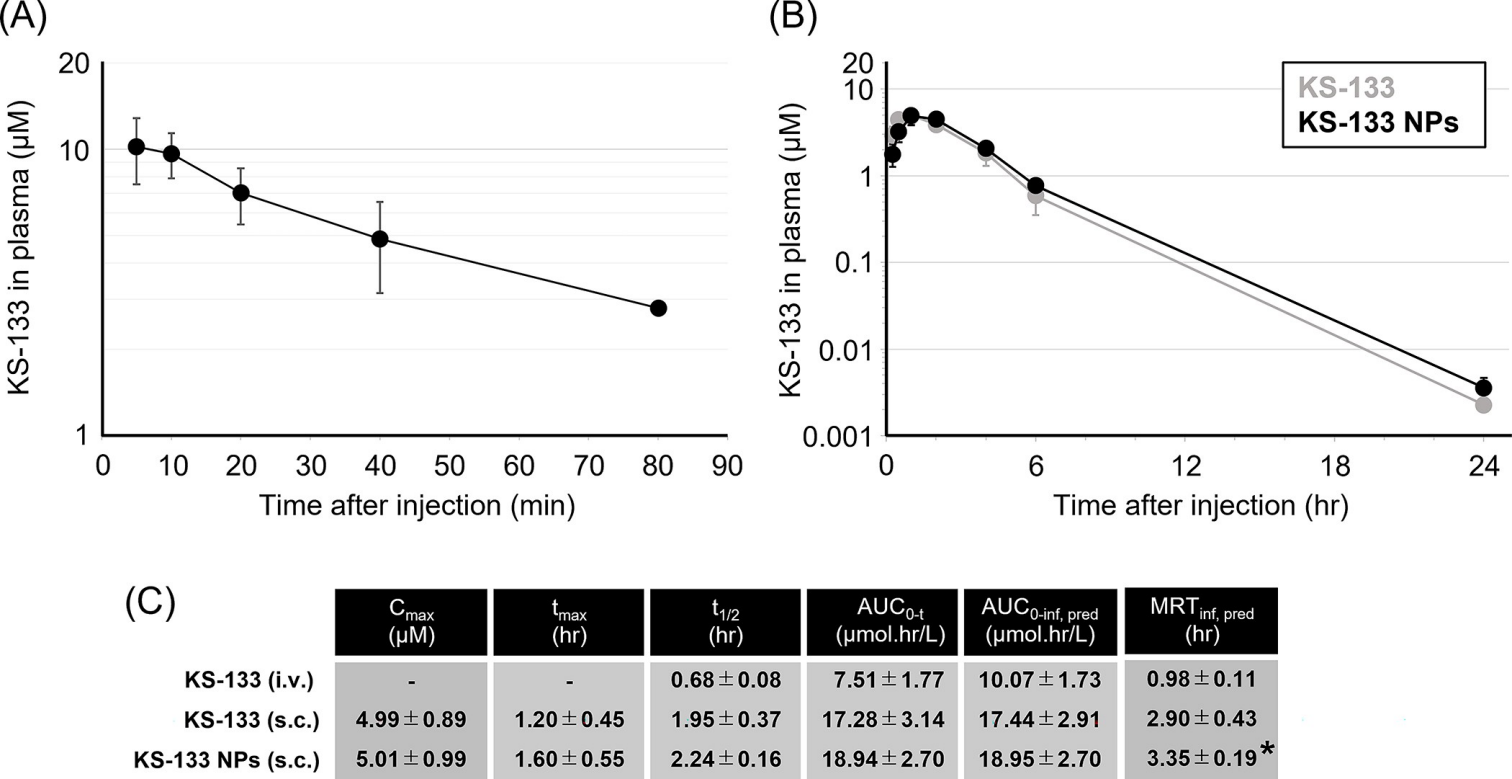
Pharmacokinetic study of KS-133 and KS-133 NPs in mice. Concentration of peptides in plasma at indicated time points after administration of peptide with or without nanoformulation are presented. (A) Intravenous administration of KS-133 (n = 3, 1 nmol/g, mean ± SD). (B) Subcutaneous administration of KS-133 and KS-133 NPs (n = 5, 1 nmol/g, mean ± SD). (C) Blood samples were collected at the indicated times. The concentration of KS-133 in plasma was determined by LC–MS/MS (mean ± SD, *p < 0.05 by Student’s *t*-test). The pharmacokinetic parameters C_max_ (μM), t_max_ (h), t_1/2_ (h), AUC_0-t_ (μmol·h/L), AUC_0-inf,pred_ (μmol·h/L), and MRT_inf,pred_ (h) were calculated as described in the “Materials and methods” section.

Pharmacokinetic studies in mice were conducted to elucidate the mechanism of the improved pharmacological effects of KS-133 upon nanoformulation. As presented in Fig 4, when KS-133 was administered intravenously or subcutaneously at 1 nmol/g, the elimination half-lives (t_1/2_) from plasma were 0.68 and 1.95 h, respectively. The longer t_1/2_ following subcutaneous administration was attributed to the absorption process from subcutaneous tissue to blood. t_1/2_ of KS-133 NPs (1 nmol/g, subcutaneous administration) was 2.24 h. There was no significant difference in the maximum drug concentration (C_max_), time to reach maximum drug concentration (t_max_), t_1/2_, area under the plasma concentration-time curve from time 0 to t (AUC_0-t_), and time 0 to infinity using predicted plasma concentration at time t (AUC_0-inf,pred_) of KS-133 with or without nanoformulation. However, the mean residence time (MRT_inf,pred_) of KS-133 NPs was 3.35 h, versus 2.90 h for KS-133 (*p* < 0.05). Thus, nanoformulation slightly improved the pharmacokinetic profiles of KS-133, which may have contributed to the improved pharmacological efficacy of the nanoformulation (Fig 3).

## Discussion

In this study, we demonstrated for the first time that KS-133, a VIPR2-selective antagonistic peptide, increases M1-type macrophage markers and decreases M2-type macrophage markers *in vitro*, exhibits anti-tumor efficacy *in vivo*, and enhances the pharmacological efficacy of an anti-PD-1 antibody. We previously reported that non-specific VIPR inhibitor, VIPhyb increased the expression of the M1-type macrophage markers *TNF*, *iNOS*, and *CXCL10* and decreased that of the M2-type macrophage marker *Mrc-1* but not of *IL-1Rn* or *CCL22* [7]. Furthermore, knockdown of *VIPR2* by siRNA decreased the expression of the M2-type macrophage markers *Mrc-1*, *IL-1Rn*, and *CCL22* without affecting M1-type macrophage marker expression [7]. In the present study, KS-133 increased the expression of the M1-type macrophage marker *iNOS* / *CXCL10* and decreased that of the M2-type macrophage marker *Mrc-1* but had no effect on *IL-1Rn* or *CCL22* expression. These data indicate that KS-133 changes the polarity of macrophages from M2 to M1 by inhibiting VIPR2.

Our data have illustrated the potent anti-tumor effects of KS-133 in a CT26 tumor-bearing mouse model. Previous studies reported that VIPR inhibition activates CD8+ cells [3–5]. However, our previous studies revealed that the administration of VIPhyb to CT26 tumor-bearing SCID mice, which have deficiencies of T and B cells, resulted in tumor growth suppression [7]. Additionally, we previously confirmed that VIPhyb does not affect proliferation of CT26 cells [7]. These data suggest that the anti-tumor effects of VIPR blockade are mediated by non-T and non-B cells system, at least in part, rather than by direct growth inhibition. In this regard, we also found that the ratio of M1/M2 macrophages in VIPhyb-administered individuals was tilted toward the M1-type and the phagocytosis of macrophages was also enhanced [7]. In the present experiment, specific VIPR2 blockade by KS-133 significantly increased M1-type macrophage marker expression and decreased M2-type macrophage marker expression. These data combined with our previous data [7] strongly support the hypothesis that the anti-tumor effect of KS-133 predominantly results from the polarity change of macrophages via direct inhibition of VIPR2. Importantly, the anti-tumor effects of KS-133 were elicited both as a single agent and in combination with an anti-PD-1 antibody. A number of human clinical trials are currently examining combinations of immune checkpoint inhibitors and anti-cancer drugs such as conventional chemotherapy drugs, metronomic chemotherapy, DNA damage repair inhibitors, and molecular targeted drugs [27]. VIPR2-selective blockade is also expected to have additive/synergistic anti-tumor effects in combination with anti-cancer drugs.

Another interesting result was the relationship between the stability and anti-cancer activity of NPs containing a VIPR2 antagonist. In general, nanoformulation technology has been developed as a DDS for nucleic acid medicines targeting intracellular genes [19, 20]. Thus, there are few reports of the application of nanoformulation technology to extracellularly targeted molecules. In addition, NPs are generally administered intravenously, and few studies have examined subcutaneous administration. The reasons for these observations are that NPs are designed to stably encapsulate the drug molecules, even in blood, until they penetrate the target cells and the transferability of subcutaneously administered NPs to blood is low. KS-133 was stably retained in NPs at low temperatures, but it was released from NPs as temperature increased. KS-133(P3S), which differs by only one amino acid from KS-133, remained encapsulated by NPs, even at 37°C. The cause of such a difference in physical properties has not been elucidated. Serine may have hydrogen bond interaction with hydroxy groups in the hydrophobic region of CrEL because it has a hydroxy group. KS-133 NPs had stronger pharmacological effects than KS-133(P3S) NPs, suggesting that the release rate of KS-133 from NPs is correlated with its anti-cancer activity. Pharmacokinetic studies revealed that the presence or absence of nanoformulation significantly affects MRT but not t_1/2_, of KS-133. The data from the pharmacokinetic study are consistent with those from the pharmacodynamic study because an increase in MRT implies an increase in the exposure time of KS-133 in body tissues. C_max_ of KS-133 NPs was approximately equal to that of non-formulated KS-133. t_max_ of KS-133 NPs tended to be higher than that of non-formulated KS-133. Therefore, it is suggested that KS-133 was released from NPs in a relatively early phase. However, it is currently unclear whether KS-133 was released from the NPs at the subcutaneous site, after transfer into blood, or both. In the pharmacokinetic test method of this study, KS-133 encapsulated within NPs and KS-133 already released from NPs could not be evaluated separately. The improved anti-tumor effect may also be attributable to an enhanced permeability and retention (EPR) effect of NPs [19, 20]. In the future, it may be necessary to label CrEL and KS-133 separately to assess the pharmacokinetics of each molecule in detail.

In conclusion, we have demonstrated that KS-133 changes the polarity of macrophages to M1 *in vitro*. Our data have also revealed that KS-133 with or without nanoformulation has anti-tumor efficacy both alone and in combination with an immune checkpoint-inhibiting anti-PD-1 antibody. In the future, KS-133 (including the nanoformulated form) is expected to be used in combination with immune checkpoint inhibitors and other drugs for treatment-resistant cancers.

## Supporting information

S1 Table (PDF)
