# Supplementary material for: The VIPR2-selective antagonist KS-133 changes macrophage polarization and exerts potent anti-tumor effects as a single agent and in combination with an anti-PD-1 antibody
Source: PLoS One. 2023 Jul 5;18(7):e0286651. doi: 10.1371/journal.pone.0286651 (PMC10321640; doi:10.1371/journal.pone.0286651)
Supplement: S1 Table — (PDF) [file pone.0286651.s001.pdf]

# The VIPR2-selective antagonist KS-133 changes macrophage polarization and exerts potent anti-tumor effects as a single agent and in combination with an anti-PD-1 antibody

Kotaro Sakamoto[1¶*], Wararat Kittikulsuth[2¶], Eijiro Miyako[3], Akumwami Steeve[2], Rika Ishimura[4], Shinsaku Nakagawa[4,5,6], Yukio Ago[6,7], and Akira Nishiyama[2]

[1]Research & Development Depertment, Ichimaru Pharcos Company Limited, 318-1 Asagi, Motosu, 501-0475 Gifu, Japan

[2]Depertment of Pharmacology, Faculty of Medcine, Kagawa University, Kagawa, 1750-1 Ikenoue, Miki-cho, Kita-gun, 761-0793 Kagawa, Japan

[3]Graduate School of Advanced Science and Technology, Japan Advanced Institute of Science and Technology, 1-1 Asahidai, Nomi, 923-1292 Ishikawa, Japan

[4]Center for Supporting Drug Discovery and Life Science Research, Graduate School of Pharmaceutical Science, Osaka University, 1-6 Yamadaoka, Suita, 565-0871 Osaka, Japan

[5]Laboratory of Biopharmaceutics, Osaka University, 1-6 Yamada-oka, Suita, 565-0871 Osaka, Japan

[6]Global Center for Medical Engineering and Informatics, Osaka University, 2-2 Yamada-oka, Suita, 565-0871 Osaka, Japan

[7]Department of Cellular and Molecular Pharmacology, Graduate School of Biomedical and Health Sciences, Hiroshima University, 1-2-3 Kasumi, Minami-ku, Hiroshima, 734-8553 Hiroshima, Japan

[¶] These two authors contributed eaqualy to this study

*Corresponding author:
Kotaro Sakamoto, Ph.D. (E-mail: sakamoto-kotaro@ichimaru.co.jp)

**Supplementary Table S1**

Analytical data of peptides in this report.

| | RP–HPLC | | Mass (Linear mode) (g/mol) | |
|---|---|---|---|---|
| Name | $T_{ret}$ (min) | Purity (%) | Calc. | Obsv.$^{\text{Voltage polarity}}$ |
| KS-133 | 10.490 | 97.98 | 1558.8 | 1558.487$^{\text{NEG}}$ |
| KS-133(P3S) | 10.191 | 96.91 | 1549.0 | 1549.805$^{\text{NEG}}$ |

Retention times and purities of peptides were characterized by RP-HPLC using SunFire C18 5 µm column (4.6 × 150 mm), at wavelength of 220 nm, under linear gradient four conditions: 20−90% acetonitrile in water containing 0.1% TFA for 20 min (1 mL/min). Molecular weights of peptides were determined by the autoflex speed MALDI-TOF mass spectrometer.
